# Changes in different parameters, lymphocyte proliferation and hematopoietic progenitor colony formation in EAE mice treated with myelin oligodendrocyte glycoprotein

**DOI:** 10.1111/jcmm.12704

**Published:** 2015-10-23

**Authors:** Vasilii B. Doronin, Taisiya A. Parkhomenko, Alexey Korablev, Ludmila B. Toporkova, Julia A. Lopatnikova, Alina A. Alshevskaja, Sergei V. Sennikov, Valentina N. Buneva, Thomas Budde, Sven G. Meuth, Irina A. Orlovskaya, Nelly A. Popova, Georgy A. Nevinsky

**Affiliations:** ^1^ Novosibirsk Medical UniversityMinistry of Public Health of Russian FederationNovosibirskRussia; ^2^ Institute of Chemical Biology and Fundamental MedicineSiberian Branch of the Russian Academy of SciencesNovosibirskRussia; ^3^ Institute Cytology and GeneticsSiberian Branch of the Russian Academy of SciencesNovosibirskRussia; ^4^ Institute of Clinical ImmunologySiberian Branch of the Russian Academy of Medical SciencesNovosibirskRussia; ^5^ Novosibirsk State UniversityNovosibirskRussia; ^6^ Institut für Physiologie IWestfälische Wilhelms‐UniversitätMünsterGermany; ^7^ Department of NeurologyWestfälische Wilhelms‐UniversitätMünsterGermany

**Keywords:** EAE model, C57BL/6 mice, catalytic antibodies, colony formation, hematopoietic progenitors, lymphocyte proliferation, apoptosis in different organs

## Abstract

Myelin oligodendrocyte glycoprotein (MOG) is an antigen of the myelin sheath, which may trigger immune cell responses and the production of auto‐antibodies in multiple sclerosis (MS). In this study, we used MOG_35‐55_‐induced experimental autoimmune encephalomyelitis (EAE), a model of human MS, to assess the production of catalytically active immunoglobulin G (IgG) antibodies or abzymes which have been shown to be present in sera of patients with several autoimmune diseases. Here, we show that IgGs from the sera of control C57BL/6 mice are catalytically inactive. During development of EAE, a specific reorganization of the immune system of mice occurred leading to a condition which was associated with the generation of catalytically active IgGs hydrolysing DNA, myelin basic protein (MBP) and MOG which was associated with increased proteinuria, changes in differentiation of mice bone marrow hematopoietic stem cells (HSCs) and an increase in proliferation of lymphocytes in bone marrow, spleen and thymus as well as a significant suppression of cell apoptosis in these organs. The strongest alterations were found in the early disease phase (18–24 days after immunization) and were less pronounced in later EAE stages (40 days after EAE induction). We conclude that a significant increase in DNase and proteolytic activities of antibodies may be considered the earliest statistically significant marker of MOG‐induced EAE in mice. The possible differences in immune system reorganizations during preclinical phases of the disease, acute and late EAE, leading to production of different auto‐antibodies and abzymes as well other changes are discussed.

## Introduction

Multiple sclerosis is a chronic demyelinating disease of the central nervous system. Its aetiology remains unclear, and the most widely accepted theory of MS pathogenesis assigns a main role to the destruction of myelin by the inflammation related to autoimmune reactions 1. Evidence supports activated CD4^+^ myelin‐reactive T cells as being major mediators of MS. Several recent findings imply an important role of B cells and auto‐antibodies (auto‐Abs) against myelin autoantigens in the pathogenesis of MS 1, 2, 3. Current evidence from animal models and clinical studies suggests that a crucial role in MS immunopathogenesis is played by auto‐Abs against myelin autoantigens, which are involved in antibody‐mediated demyelination 3, and by auto‐Abs against oligodendrocyte progenitor cell protein, which could block remyelination by eliminating or impeding these cells 4. An important dual role of auto‐Abs is suggested: they may not only be harmful in lesion formation but also potentially beneficial in lesion repair 2. Elevated Abs levels and oligoclonal IgG in the cerebrospinal fluid (CSF) as well as clonal B‐cell accumulation in the CSF and lesions of MS patients are among the main lines of evidence for the involvement of a humoural response in demyelination 5.

Abs against transition states of chemical reactions and natural abzymes catalysing more than 100 distinct chemical reactions are novel biological catalysts that have attracted much interest over the last years (for review see Ref. 6, 7, 8, 9). Natural catalytic abzymes hydrolysing DNA, RNA, polysaccharides, oligopeptides and proteins have been revealed in the sera of patients with many autoimmune and viral diseases (for review, see Ref. 9, 10, 11, 12, 13, 14).

Levels of abzymes with DNase and RNase activities in healthy human beings and animals are usually close to the detection limit of common methods 9, 10, 11, 12, 13, 14. In the sera of healthy mammals auto‐Abs to different antigens, including DNA and various proteins, are detectable but their titres vary significantly 15, 16 and all of them are catalytically inactive 10, 11, 12, 13, 14. We have shown that the appearance of abzymes specific for various substrates is one of the earliest and characteristic signs of autoimmune reactions in a number of autoimmune diseases [systemic lupus erythematosus (SLE), Hashimoto's thyroiditis, polyarthritis and MS] and viral diseases with strong immune system disturbances (autoimmune deficiency syndrome, hepatitis and tick‐borne encephalitis) 10, 11, 12, 13, 14. According to our data, the catalytic activity of nuclease abzymes is usually very easily detectable at the beginning of autoimmune diseases, when concentrations of Abs to DNA or other autoantigens are not yet significantly increased, and correspond to levels in healthy donors 10, 11, 12, 13, 14.

Similar to SLE, high‐affinity anti‐DNA Abs have been recently identified as a major component of intrathecal IgGs in the brain and CSF cells of MS patients 17. It was shown that homogeneous IgGs from sera and CSF of MS patients were active in the hydrolysis of DNA, RNA and polysaccharides 18, 19, 20, 21. Although only 18% and 53% of MS patients demonstrated increased concentrations of Abs to native and denatured DNA respectively. Compared with healthy donors, DNase abzymes were found in 80–90% of MS patients 18, 19. Since the DNase abzymes of MS patients 22 and SLE patients 23, are cytotoxic and induce apoptosis, they may play an important role in the pathogenesis of both disorders.

It has been recently shown that MBP‐hydrolysing activity is an intrinsic property of IgGs, IgMs and IgAs from the sera of MS patients 24, 25, 26, 27, and the specific sites of neural antigen cleaved by abzymes have been identified 27. Recognition and degradation of MBP peptides by serum auto‐Abs has been confirmed to be a novel biomarker for MS 28. In MS, anti‐MBP abzymes with protease activity can attack MBP of the myelin‐proteolipid sheath of axons. The established MS drug glatiramer acetate (Copaxone^®^) appears to be a specific inhibitor of MBP‐hydrolysing abzyme activity 27, 29. Consequently, these abzymes may play an important harmful role in MS pathogenesis.

MRL‐lpr/lpr mice spontaneously developing a SLE‐like disorder are characterized by marked hypergammaglobulinemia, production of numerous auto‐Abs, circulating immune complexes, glomerulonephritis and severe lymphadenopathy. A mutation in the *lpr* gene of these mice leads to a deficit in functional Fas ligand and dysregulation of apoptosis in homozygotes 28, 29, 30. As a result, the mice develop a SLE‐like phenotype, including accumulation of double‐negative T cells (CD4^−^ CD8^−^ B220^+^ TCR^+^) in peripheral lymphoid organs. Systemic lupus erythematosus is one of several autoimmune diseases with an increased level of anti‐DNA Abs. Many SLE anti‐DNA Abs are directed against histone‐DNA nucleosomal complexes, appearing as a result of internucleosomal cleavage during apoptosis. Apoptotic cells are the primary source of antigens and immunogens in SLE resulting in the recognition, perception, processing and/or presentation of apoptotic auto‐antigens by antigen‐presenting cells, and can cause autoimmune processes 31. Different abzymes were obtained with a dramatically higher incidence in autoimmune mouse strains than in conventionally used control mouse strains 32, 33. The sera of autoimmune‐diseased MRL‐lpr/lpr mice contain abzymes hydrolysing DNA, adenosine 5′‐triphosphate (ATP) and oligosaccharides with high catalytic activities 34, 35, 36.

An ever‐increasing number of observations suggest that autoimmune diseases originate from defects in HSCs 37. It has recently been shown that the specific reorganization of the immune system during the spontaneous development of a profound SLE‐like pathology in MRL‐lpr/lpr mice is associated with changes in the differentiation profile of bone marrow HSCs and the level of lymphocyte proliferation in combination with the production of DNase, ATPase and amylase abzymes 34, 35, 36. Immunization of healthy mice with DNA also leads to the production of Abs with DNase activity; however, it is only associated with increased lymphocyte proliferation and suppression of lymphocyte apoptosis in different organs (especially the spleen), but not with a change in the differentiation of bone marrow cells 34, 35, 36. In consequence, it was suggested that MS patients may reveal similar changes in the differentiation profile of bone marrow HSCs and the level of lymphocyte proliferation, and, as a consequence, MS patients can produce not only Abs to MBP and DNA, but also specific abzymes. It has been recently shown that the CSF of MS patients contain abzymes with DNase‐ and MBP‐hydrolysing activity, and that the specific activities of these Abs in the CSF are approximately 50‐ to 60‐fold higher than those in the corresponding sera 38, 39.

To determine a possible role of abzymes in the EAE disease course, it was interesting to analyse the effect of MOG immunization on the differentiation profile of bone marrow HSC and the level of lymphocyte proliferation which may be associated with the production of MBP‐ and DNA‐hydrolysing abzymes. Here we have assessed, for the first time, the correlation of the relative activities of mouse IgGs in the hydrolysis of MBP, MOG and DNA with some biochemical markers of EAE pathology (proteinuria, Abs titres to MBP, MOG and DNA) as well as with changes in the differentiation profile and the level of proliferation of bone marrow HSCs, proliferation and apoptosis of lymphocytes in different organs during various time‐points before and after mice treatment with MOG.

## Materials and methods

### Reagents

If not stated otherwise chemicals, proteins, Protein G‐Sepharose and the Superdex 200 HR 10/30 column were purchased from Sigma or GE Healthcare. We used purified human MBP containing 18.5 kD from RCMDT (Moscow, Russia), MOG_35‐55_ from EZBiolab, *Bordetella pertussis* toxin (*Mycobacterium tuberculosis*) from Native Antigen Company (Oxfordshire, UK). These preparations were free from lipids, oligosaccharides, nucleic acid and other possible contaminants.

### Experimental animals

C57BL/6 inbred mice and control non‐autoimmune BALB/c and (CBAxC57BL)F1 (CBA), 3 months aged, were housed in colonies under the same standard pathogen‐free conditions, including a system for protection from bacterial and viral infections, at the Institute of Cytology and Genetics mouse breeding facility. All experimental procedures with mice were conducted in accordance with protocols of Bioethical Committee of Institute of Cytology and Genetics corresponding to protocols of Bioethical Committee of the Siberian Branch of the Russian Academy of Sciences and the recommendations of the European Committee for the humane principles of work with experimental animals (European Communities Council Directive 86/609/CEE.) Bioethical Committee of Institute of Cytology and Genetics has approved our study in accordance with European Communities Council Directive 86/609 guidelines.

### Immunization of mice

Immunization of mice with MOG was performed with MOG and Pertussis toxin according to previously published protocol 40. On day 1 (zero time), mice were immunized by injection of 10 μg of MOG per mouse in the back, two times in the left and right side using 20 μl of Freund's complete adjuvant containing Pertussis toxin (400 ng/mouse; *M. tuberculosis*). The next day an additional 20 μl of Pertussis Toxin (400 ng/mouse) was injected in a similar way. The relative weight of mice and degree of proteinuria (relative concentration of total protein in the urine, mg/ml) were analysed as before 36. Protein concentration in the urine was measured using the Bradford assay with a bovine serum albumin standard. For several types of analyses, we used a small volume of blood (20–40 μl) collected every 6–12 days during the 0–40 days period of the experiment from the mouse eyes. In addition, for other experiments including purification of Abs and analysis of their enzymatic activity 0.7–1 ml of the blood were collected after decapitation using standard approaches.

### ELISA of anti‐protein and anti‐DNA Abs

Anti‐MOG and anti‐MBP antibody concentrations were measured by ELISA (plasma was diluted 50‐fold). The concentration of serum anti‐DNA Abs was determined using standard ELISA plates with immobilized double‐stranded DNA (plasma was diluted 100‐fold) as described in 36. After a consecutive treatment of samples with the blood serum and rabbit‐specific antimouse Abs conjugated with horseradish peroxidase, the reaction mixtures were incubated with tetramethyl benzidine and H_2_O_2_. The reaction was stopped with H_2_SO_4_ and the optical density (A_450_) of the solutions was determined using a Uniskan II plate reader (MTX Lab Systems, Vienna, VA, USA). The relative concentrations of anti‐MOG and anti‐DNA Abs in the samples were expressed as a difference in the relative absorption at 450 nm between experimental and control samples; controls with MOG or DNA, but without serum samples and with Abs not interacting with MOG or DNA, gave the same results.

### IgG purification

Electrophoretically and immunologically homogeneous mouse IgGs were obtained by sequential chromatography of the serum proteins on Protein G‐Sepharose and following fast protein liquid chromatography (FPLC) gel filtration as described previously 24, 25, 26, 34, 35, 36. To protect Abs preparations from bacterial and viral contamination, they were filtered through Millex syringe‐driven filtre units (0.2 μm) and kept in sterilized tubes. Incubation of standard bacterial medium with stored Abs preparations did not lead to the formation of colonies. SDS‐PAGE analysis of the Ab fractions for homogeneity under non‐reducing conditions was done in 4–15% gradient gels; for polypeptide separation, it was performed in a reducing gel (0.1% SDS and 10 mM dithiothreitol), and the polypeptides were visualized by silver staining as previously described 24, 25, 26, 34, 35, 36. To exclude possible artefacts because of hypothetical traces of contaminating enzymes, IgGs were separated by SDS‐PAGE and their protease and nuclease activities were detected using a gel assay as stated earlier 24, 25, 26, 34, 35, 36. These activities were only revealed in the band corresponding to intact IgGs and there were no other peaks of proteins, protease or DNase activities.

### DNA‐hydrolysing activity assay

DNase activity was analysed similar to published methods 36. The reaction mixture (20 μl) for analysis of IgG DNase activity contained 20 μg/ml supercoiled (sc) pBluescript, 20 mM NaCl, 5 mM MgCl_2_, 1 mM ethylenediaminetetraacetic acid (EDTA), 20 mM Tris‐HCl, pH 7.5, and 0.03–0.2 mg/ml of Abs, and was incubated for 2–12 hrs at 37°C. Cleavage products were analysed by electrophoresis in 0.8% agarose gels. Ethidium bromide‐stained gels were photographed and the films were scanned. The activities of IgG preparations were determined from the scans (Gel‐Pro Analyzer v9.11; Media Cybernetics, Rockville, MD, USA) as a relative percentage of DNA corresponding to an initial band of scDNA and its relaxed form, a distribution of DNA between these bands in the case of the control experiment (incubation of pBluescript in the absence of Abs) was taken into account. All measurements (initial rates) were taken within the linear regions of the time course (15–40% of DNA hydrolysis) and a complete transition of scDNA to nicked DNA was taken as 100% of the activity. If the activity was low (<5–10% of scDNA disappearance), the incubation time was increased to 3–12 hrs, depending on the sample. If the degradation of scDNA exceeded 50%, the concentration of Abs was decreased 2‐ to 100‐fold, depending on the sample analysed. Finally, the RAs were normalized to the same conditions.

### Protease activity assay

The reaction mixture (10–40 μl) for analysis of MBP‐ or MOG‐hydrolysing activity of IgGs, containing 20 mM Tris‐HCl (pH 7.5), 0.5–0.7 mg/ml MBP or MOG and 0.01–0.2 mg/ml of IgGs was incubated for 2–18 hrs at 37°C. MBP and MOG cleavage products were analysed by SDS‐PAGE in 12% or 4–15% gradient gels with Coomassie R250 staining. The gels were scanned and quantified using GelPro v3.1 software (Media Cybernetics, Rockville, MD, USA). The activities of IgG preparations were determined as a decrease in the percentage of initial MBP or MOG converted to different hydrolysed forms compared with control MBP or MOG incubated without Abs. All measurements (initial rates) were taken under the conditions of the pseudo‐first order of the reaction within the linear regions of the time course (15–40% of MBP) and dependencies of MBP hydrolysis on IgG concentration.

### Analysis of bone marrow progenitor cells in culture and lymphocyte proliferation

Bone marrow was flushed out of the mouse femurs. To assess the colony‐forming ability, of bone marrow cells, 2 × 10^4^ cells (four dishes per mouse) were cultured in a standard methylcellulose‐based M3434 medium for mouse cells (StemCell Technologies, Vancouver, British Columbia, Canada) containing stem cell factor, erythropoietin, interleukin (IL)‐3 and IL‐6. Colonies [granulocytic‐macrophagic colony‐forming unit (CFU‐GM), erythroid burst‐forming unit – early erythroid colonies (BFU‐E), erythroid burst‐forming unit – late erythroid colonies (CFU‐E), and granulocytic‐erythroid‐megacaryocytic‐macrophagic colony‐forming unit (CFU‐GEMM)] were scored after 14 days of incubation at 37°C and 5% CO_2_ in a humidified incubator as described previously 34, 35, 36, 41.

All *in vitro* assays of lymphocyte proliferation were performed as described earlier 41. For an estimation of proliferative ability, cells (10^6^/ml) isolated from bone marrow, spleen, lymph nodes and thymus were cultivated in 96‐well flat‐bottom plates (TPP, Trasadingen, Switzerland) using RPMI‐1640 medium containing 10% of foetal calf serum, 2 mM L‐glutamine, 10 mM HEPES (4‐(2‐hydroxyethyl)‐1‐piperazineethanesulfonic acid) buffer, 0.5 mM 2‐mercaptoethanol, 80 μg/ml gentamicin and 100 μg/ml benzylpenicillin. After a 64‐hr incubation, 15 μl MTT [5 mg/ml; tetrazolium dye MTT 3‐(4,5‐dimethylthiazol‐2‐yl)‐2,5‐diphenyltetrazolium bromide] solution was added to every hole and plates were incubated for another 4 hrs at 37°C. Plates were then centrifuged at 1200 r.p.m. (300 g) for 10 min. and solutions were removed. After the addition of DMSO (200 μl), the precipitated cells were resuspended and incubated for 15 min. at room temperature in darkness. The analysis of cells was carried out spectrophotometrically at 492 nm.

### Apoptosis assay

Cell apoptosis assay was performed according to previous studies 41, 42. Cells (1 × 10^6^/ml) were incubated for 48 hrs at 37°C using RPMI‐1640 medium containing 10% foetal calf serum, 2 mM L‐glutamine, 10 mM HEPES buffer, 0.5 mM 2‐mercaptoethanol, 80 μg/ml gentamicin and 100 μg/ml benzylpenicillin (5% CO_2_). After incubation, cells were washed with 2 ml PBS containing 0.02% EDTA and 0.1% NaN_3_ 41, 42. The cells were then incubated using 500 μl solution containing 0.1% Triton X‐100, 250 μg/ml RNase and 50 μg/ml propidium iodide (PI) for 20 min. at 37°C. PI fluorescence of individual nuclei was measured using BD FacsVerse flow cytometer (BD Biosciences, San Jose, CA, USA). The results are reported as the percentage of hypodiploid (fragmented) nuclei reflecting the fraction of apoptotic cells.

### Statistical analysis

The results are reported as the mean ± S.D. of at least 3–4 independent experiments for each mouse, averaged over seven different animals. Differences between the samples were analysed by Student's *t*‐test, *P* ≤ 0.05 was considered to be statistically significant.

## Results

### Experimental groups of mice

In MOG‐induced EAE mice first clinical symptoms appear at 5–7 days after immunization, while the maximum stage of the disease is usually manifested at 14–16 days after immunization 40, 43.

In this study, we have used three experimental groups of C57BL/6 and CBA mice:

1. control non‐autoimmune (CBA × C57BL) F1 or CBA mice,
2. untreated control C57BL/6 mice and
3. MOG‐immunized C57BL/6 mice.

**Figure 1:**
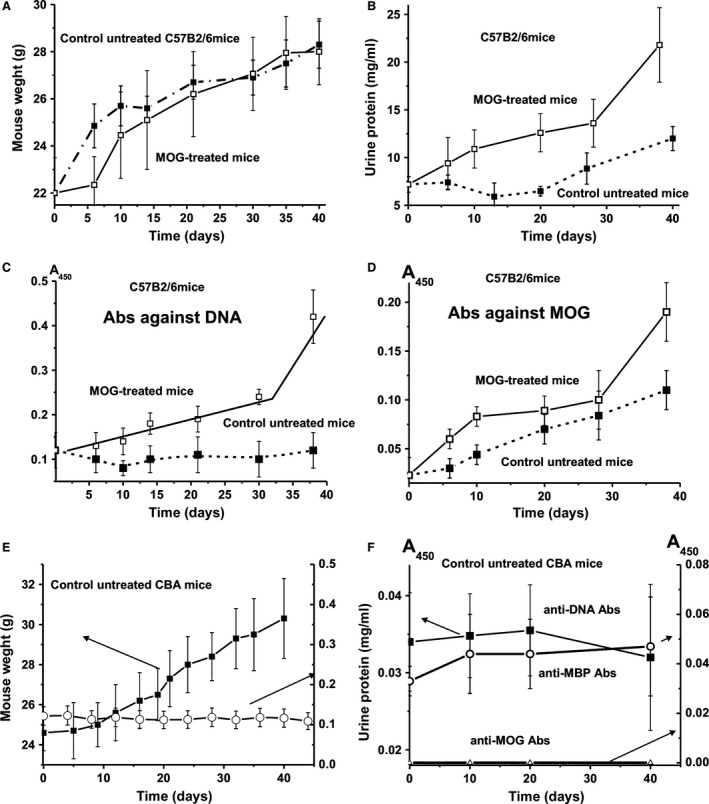
In time changes in weight of C57B2/6 mice (**A**), relative concentration of urine protein (**B**), titres of Abs to DNA (**C**) and Abs to MOG (**D**) in mice untreated and treated with MOG. Anti‐MOG and anti‐MBP antibody concentrations in the sera of C57B2/6 mice were measured by ELISA (plasma was diluted 50‐fold). The concentration of plasma anti‐DNA Abs was determined using standard ELISA plates with immobilized double‐stranded DNA (plasma was diluted 100‐fold). In time changes in weight and in relative concentration of urine protein of control CBA mice (**E**), titres of Abs to DNA, MBP and MOG (**F**). Plasma of CBA mice was diluted 10‐fold and then the A_450_ values obtained were recalculated to 50‐ (MOG and MBP) and 100‐fold (DNA) dilution respectively.

We analysed possible changes in the relative weight, different immunological and biochemical parameters at 3 months of age (zero time; control) and MOG‐treated C57BL/6 mice during consecutive 40 days (Fig. 1A). After 6 days but not at later stages, an average weight loss was found in treated compared to untreated control mice (Fig. 1A).

In other models of autoimmunity including MRL‐lpr/lpr, the appearance of pronounced visual symptoms usually correlates well with proteinuria (≥3 mg/ml concentration of protein in urine) 34, 35, 36. Control non‐autoimmune BALB and CBA mice observed for at least 7 months demonstrated no proteinuria (0.1–0.12 mg/ml) 34, 35, 36. Healthy MRL‐lpr/lpr autoimmune mice before development of spontaneous pronounced SLE are usually characterized by relatively low proteinuria (0.38 mg/ml) 34, 35, 36. At the same time, C57BL/6 mice are usually characterized by a significantly higher level of proteinuria (up to 10–12 mg/ml) 44. We have analysed the time‐dependent change in proteinuria for immunized and untreated C57BL/6 mice (Fig. 1B). In seven mice of the control group the average urine protein concentration was 7.2 ± 0.8 mg/ml at time zero (3 months of age), remained nearly stable until day 20 and increased to 12 ± 0.8 mg/ml at 40 days. In seven mice treated with MOG, a steady increase in proteinuria was observed (day 0: 7.2 ± 0.8 mg/ml; day 30: 13.6 ± 2.5 mg/ml; day 40: 21.8 ± 3.8 mg/ml). The increase after 40 days became significantly different from control animals (Fig. 1B).

### Determination of the relative content of anti‐DNA and anti‐MOG Abs

The sera of MS patients have been shown to contain anti‐MBP 24, 25, 26, 27, 28 and anti‐DNA 18, 19 Abs. The relative concentration of anti‐DNA Abs in the case of CBA and Balb mice during 2–7 months of age and healthy MRL‐lpr/lpr (2–3 months of age) is typically in the range of 0.03–0.04 A_450_ units 36. Using the same test, we have analysed the relative concentration of anti‐DNA Abs in the sera of C57BL/6 mice. At 3 months of age the average concentration of anti‐DNA Abs of C57BL/6 mice was 0.12 ± 0.04 A_450_ units (Fig. 1C) and remained rather stable during 40 days of the analysis. At the same time, after immunization with MOG, the relative average titre of anti‐DNA Abs nearly linearly increases during ~30 days to 0.24 ± 0.017 A_450_ units (~2‐fold) and then at day 40 statistically significantly increased to 0.42 ± 0.06 A_450_ units (Fig. 1C). Thus, the treatment of C57BL/6 mice with MOG stimulated the formation of anti‐DNA Abs, which is a specific characteristic not only for SLE and MS patients, but also several other autoimmune diseases (see above).

Figure 1D demonstrates time‐dependent changes in anti‐MOG antibodies in the sera of non‐treated and treated C57BL/6 mice. The relative average concentration of anti‐MOG antibodies for seven untreated mice significantly (*P* ≤ 0.05) increased during ~40 days approximately 4.8‐fold from 0.023 to 0.11 A_450_ units in nearly linear fashion. In the case of immunized mice, there was an initial significant increase in the concentration of anti‐MOG antibodies until day 10 by a factor of ~3.9, followed by a plateau phase and a significant steep increase between day 30 and day 40. The level of anti‐MOG antibodies of the treated C57BL/6 mice was 8.3‐fold and 1.7‐fold higher than that of the untreated mice at the beginning of the measurement and at day 40 respectively.

**Figure 2:**
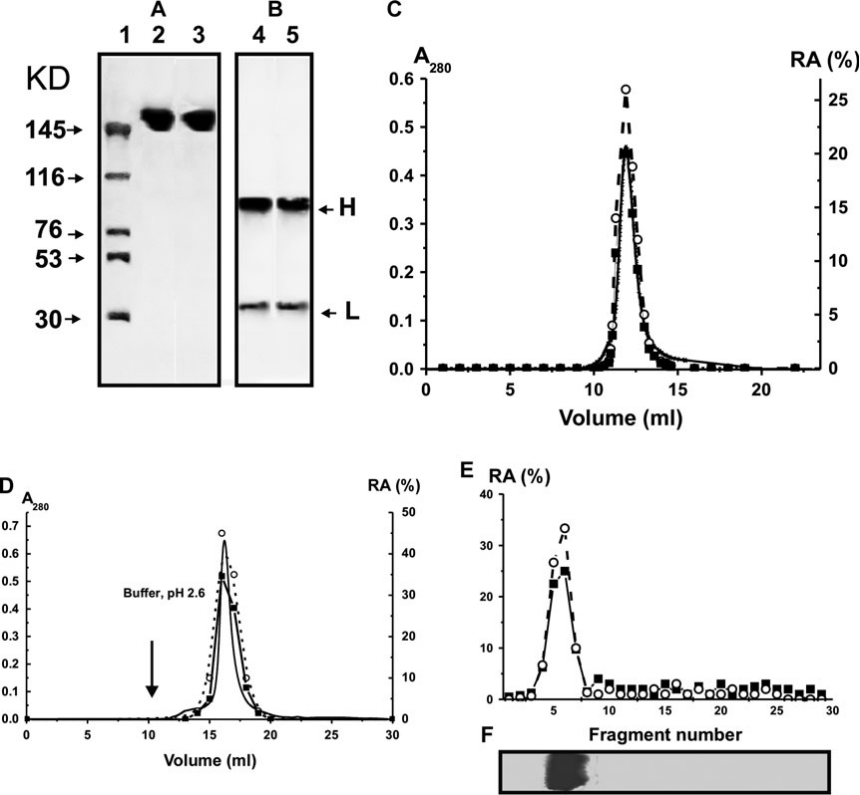
SDS‐PAGE analysis of the homogeneity of untr‐IgG_mix_ and tr‐IgG_mix_ (7 μg) under non‐reducing (**A**, lanes 2 and 3, respectively) and reducing (**B**, lanes 4 and 5) conditions followed by silver staining; the arrows (**A**, lane 1) indicate the positions of molecular mass markers. FPLC gel filtration of tr‐IgG_mix_ on a Superdex 200 column in an acidic buffer (pH 2.6) after Abs incubation in the same buffer (**C**), affinity chromatography on Sepharose bearing rabbit IgGs against mouse IgGs (**D**) and SDS‐PAGE isolation of tr‐IgG_mix_ (**E** and **F**): (—), absorbance at 280 nm (A_280_); relative activity (RA) of in the hydrolysis of DNA (○) and MBP (■).The relative DNase‐ and MBP‐hydrolysing activity (%) was revealed using the extracts of 2–3 mm fragments of one longitudinal slice of the gel (**E**). (**F**) It shows the position of IgGs after electrophoresis. A complete hydrolysis of substrates for 24 hrs was taken for 100% (**C**–**E**). The error in the initial rate determination from two experiments in each case did not exceed 7–10%. For other details, see Materials and methods.

Figure 1E demonstrates in time changes in the weight and in the relative concentration of urine protein of control non‐autoimmune prone CBA mice. One can see that the relative change in weight of CBA mice is comparable with that for C57BL/6 mice. At the same time, there is no remarkable change in the urine protein concentration for CBA mice during 40 days of the experiment (Fig. 1E). The relative concentrations of anti‐DNA Abs (A_450_) in the plasmas of untreated CBA mice also did not change remarkably during 40 days (Fig. 2F). Similar results concerning urine protein and anti‐DNA Abs concentrations were obtained earlier for untreated CBA and Balb mice 34, 35, 36.

At 3 month of age (zero time of the experiment), the relative levels of anti‐DNA and anti‐MBP Abs of CBA mice plasmas on average were approximately 3.5‐ to 4.0‐fold lower than those for C57BL/6 mice. Interestingly, C57BL/6 untreated mice at 3 months of age demonstrated reliable detectable level of anti‐MOG Abs (0.023 A_450_), which increases by a factor of approximately 4.8–40 days (0.11 A_450_) of the experiment (Fig. 1B). The plasmas of the control CBA mice did not contain anti‐MOG Abs, even after 10‐fold of the plasmas dilution there were no detectable difference in the relative absorption at 450 nm between experimental and control samples with MOG, but without serum samples. These data indicate that in contrast to EAE C57BL/6 mice, non‐autoimmune CBA mice do not in time demonstrate any detectable changes in the analysed parameters associated with the development of autoimmune processes. In addition, the data indicate that MOG treatment accelerates the time‐dependent increase in anti‐MOG and anti‐DNA antibodies in C57BL/6 mice.

### Determination of the relative proteolytic and DNase activities of antibodies

Blood of seven untreated C57BL/6 control mice was collected at day 0. Blood of untreated and MOG‐treated C57BL/6 mice was then collected at 6–40 days after immunization. To search for abzymes, IgGs were purified from the plasma of individual animals by chromatography using Protein G‐Sepharose under special conditions to remove non‐specifically bound proteins as before 24, 25, 26, 34, 35, 36. Then, IgGs were subjected to FPLC gel filtration. For analysis of protease and DNase activities of Abs, we used individual preparations from different mice and mixtures of equal amounts of electrophoretically homogeneous IgGs from the sera of seven untreated mice (untr‐IgG_mix_; 3 months of age) and 21 MOG‐treated mice (tr‐IgG_mix_; three groups of seven mice at 14–40 days after treatment). The homogeneity of typical 150‐kD untr‐IgG_mix_ and tr‐IgG_mix_ was confirmed by SDS‐PAGE with silver staining, which showed a single band under non‐reducing conditions (Fig. 2A) and two bands corresponding to the H and L chains after antibody reduction (Fig. 2B). It was shown, that IgGs from control non‐autoimmune CBA and BALB mice (3–7 months of age) do not demonstrate detectable activity in the hydrolysis of DNA, MOG and MBP, while IgGs even from untreated C57BL/6 mice were catalytically active in hydrolysis of these substrates (see below).

Application of a set of strict criteria worked out previously 10, 11, 12, 13, 14 allowing us to conclude that the observed proteolytic and DNase activities are intrinsic properties of C57BL/6 mice IgGs and are not because of co‐purifying enzymes.

The most important criteria are: (*i*) electrophoretic homogeneity of IgGs (Fig. 2A and B); (*ii*) FPLC gel filtration of tr‐IgG_mix_ under conditions of acidic shock (pH 2.6) did not lead to a disappearance of the activities, and the peaks of the protease and DNase enzymatic activities tracked exactly with IgGs (Fig. 2C); (*iii*) complete absorption of the activities by Sepharose bearing Abs against mouse IgG light chains, leading to a disappearance of the catalytic activities from the solution, and its recovery with acidic buffer (pH 2.6; Fig. 2D). To exclude possible artefacts because of potential traces of contaminating enzymes, the tr‐IgG_mix_ preparation was also analysed by SDS‐PAGE and their protease and DNA‐hydrolysing activities were detected after extraction of proteins from the separated gel slices (Fig. 2E). Both enzymatic activities were revealed only in the size of intact IgGs. Since SDS dissociates any protein complexes, and the electrophoretic mobility of known low molecular mass canonical proteases and DNases (28–32 kD) cannot coincide with that of intact IgGs (150 kD), the detection of proteolytic and DNase activities in the gel region corresponding only to intact IgGs together with the absence of any other bands of the activities or protein (Fig. 2), provides direct evidence that C57BL/6 mice IgGs possess both activities.

**Figure 3:**
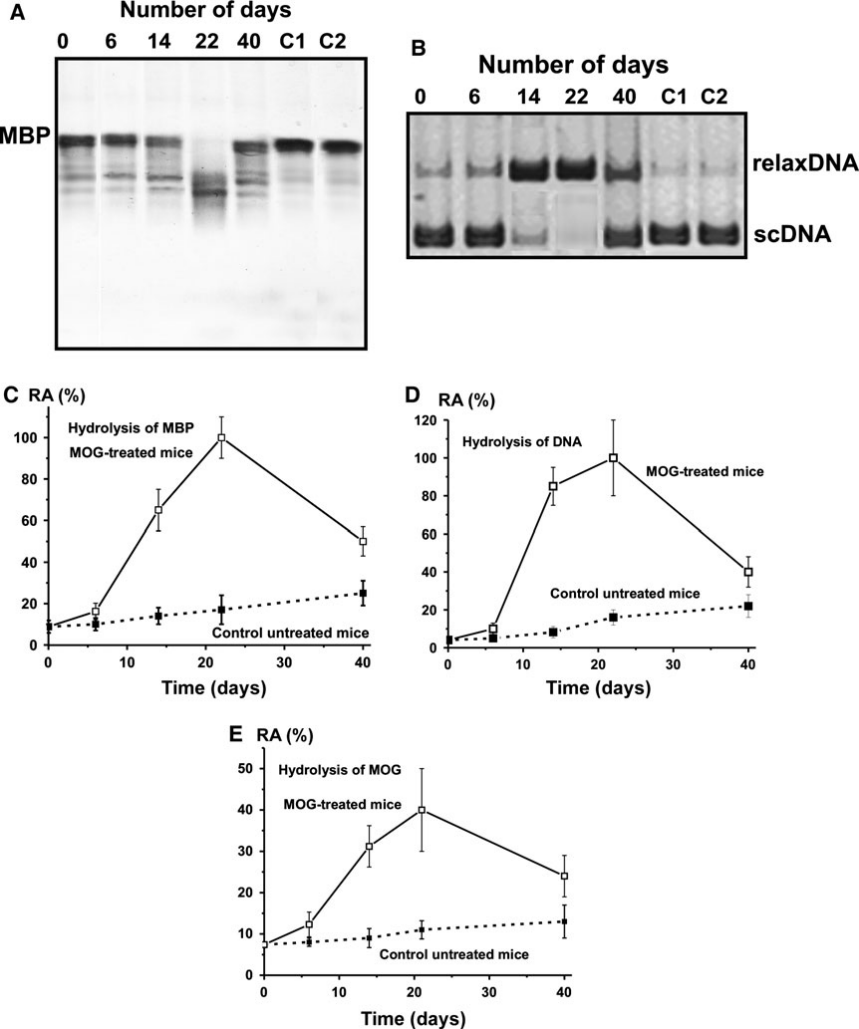
Analysis of the relative MBP‐hydrolysing activity by SDS‐PAGE (**A**) and DNase activity by agarose electrophoresis (**B**) of IgGs from the blood plasma of mice treated with MOG at 0–40 days after their immunization; equimolar mixtures of IgGs of control group of untreated mice (lane 0; 7 mice) and of the MOG‐treated groups of mice (each group of seven mice) after 6, 14, 22 and 40 days after the treatment. Lanes C1 and C2 correspond, respectively, to these substrates incubated in the absence of IgGs and in the presence of Abs from control CBA mice. MBP (0.5 mg/ml) and scDNA (20 μg/ml) were incubated in the absence or in the presence of 0.1 mg/ml IgGs for 9 hrs. In time changes in the relative average activity of IgGs from the blood plasma of untreated and MOG‐treated mice in the hydrolysis of MBP (**C**), DNA (**D**) and MOG (**E**); average relative activity (RA) of IgGs corresponding to seven individual mice of each group is given. The error in the initial rate determination from two experiments in the case of every mouse of each group did not exceed 7–10%. For other details, see Materials and methods.

Next, we measured the relative proteolytic and DNase activities (RAs) of equimolar mixtures of IgGs of untreated control groups (each group of seven mice) and of the MOG‐treated groups (each group of seven mice) after 6, 14, 22 and 40 days of the treatment. Figure 3 demonstrates the results of the RAs of these IgG mixtures in the hydrolysis of MBP (A) and DNA (B). To quantify the MBP‐, MOG‐ and DNA‐hydrolysing activities of IgGs of different groups, we determined a concentration for each individual IgG preparation that converted MBP or MOG to their corresponding hydrolysis products (no more than 40%) and scDNA to relaxed plasmid DNA without formation of linear or fragmented DNA (no more than 40% of initial DNA). The relative efficiency of MBP, MOG and DNA cleavage was calculated from the relative percentage of MBP, MOG and DNA in the bands of initial non‐hydrolysed and hydrolysed substrates. A relative amount of control substrates incubated in the absence of IgGs (or with Abs from healthy CBA mice) corresponding to two bands of initial and hydrolysed substrate was taking into account. Since all measurements (initial rates) were taken within the linear regions of the time courses and Abs concentration curves, the measured RAs for IgGs were normalized to standard conditions and a complete transition of MBP and MOG to their products or scDNA to its relaxed form was taken as 100% of analysed activity. The data on changes in average activities for seven mice of each group in time are summarized on Figure 3C–E.

During 40 days of the experiment non‐treated control mice demonstrated nearly linear statistically significant average increase in MBP‐ (from 8.8% to 25%), MOG‐ (from 7.4% to 13%) and DNase (from 4.1% to 22%) activities (Fig. 3). Six days after treatment, a time‐point corresponding to the first appearance of symptoms of EAE (usually 5–7 days after immunization 40, 43) all activities increased in MOG‐treated mice at about 1.6‐ to 1.9‐fold in comparison with those for non‐treated mice (Fig. 3). According to the literature, the maximum stage of the disease is usually manifested at 14–18 days after immunization of the mice with MOG 40. The maximal statistically significant increase in proteolytic and DNase activities of IgGs from MOG‐treated mice was observed at 22 days after immunization and they were significantly higher than those at time‐point zero: 5.5‐fold MOG, 11.4‐fold MBP and 24‐fold DNA respectively (Fig. 3). At 22 days after the treatment, the activities of IgGs from treated mice were significantly 3.6‐, 5.9‐ and 6.3‐fold higher in the hydrolysis of MOG, MBP and DNA, respectively, in comparison with these values for untreated mice (Fig. 3). At late EAE stages (40 days after treatment), the RAs of IgGs in the hydrolysis of MOG, MBP and DNA were markedly decreased but still were significantly higher (1.8‐ to 3.0‐fold) compared to untreated mice (Fig. 3). Figure 2 demonstrates a significant increase in the total titres of Abs against DNA and MOG at 20–40 days after mice treatment with MOG. These findings indicate that the transition from early to late EAE stages can lead to a decrease in the production of abzymes with different enzymatic activities because of a switch in the mouse immune system towards the synthesis of antibodies without catalytic activity. In consequence a significant decrease in the relative specific activities of Abs at 40 days in the hydrolysis of MOG, MBP and DNA was found (Fig. 3). These data are in agreement with an increase in RAs of abzymes against different antigens at the onset of autoimmune diseases and a decrease in their activities during remissions (for review, see Ref. 9, 10, 11, 12, 13, 14).

### Colony formation of hematopoietic progenitors

**Figure 4:**
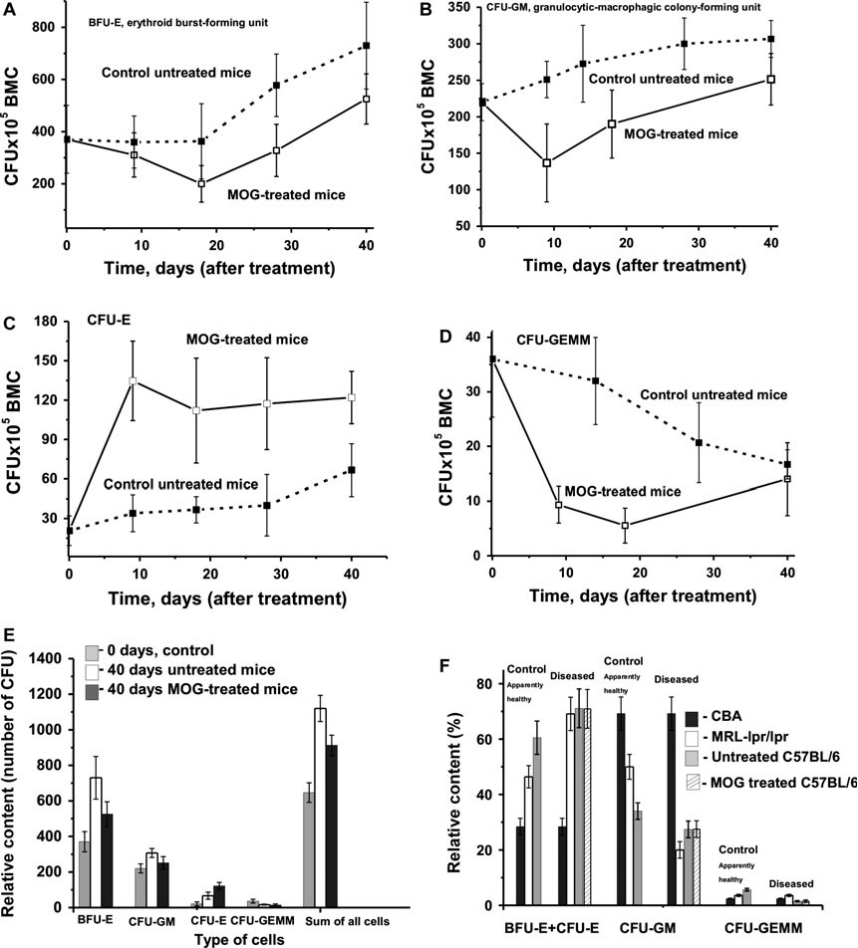
In time changes of an average relative content of colony‐forming units (CFU) of bone marrow progenitor colonies (BMC) of different type in the case of untreated and MOG‐treated mice; average number of the colonies corresponding to seven mice of each group is given and types of progenitor colonies are shown on **A**–**D**. (**E**) It demonstrates the relative profile of differentiation of bone marrow progenitors (relative number of colonies of different type) at the beginning of experiment (zero time, control) and after 40 days for untreated and MOG‐treated mice. In addition, the relative content of all type of progenitor colonies (their sum) is given. The relative content (%) of total erytroid cells (BFU‐E+ CFU‐E), CFU‐GM and CFU‐GEMM colonies in the case of healthy CBA, conditionally healthy MRL‐lpr/lpr and C57BL/6 mice at 3 months of age, after development of respectively EAE and SLE is shown (**F**). For C57BL/6 mice the relative contents of progenitor colonies after spontaneous and MOG‐stimulated development of EAE are given. For other details, see Materials and methods.

In control untreated mice, the average number of BFU‐E (~2.0‐fold), CFU‐E (~3.2‐fold) and CFU‐GM (~1.4‐fold) increased during the 40 day experimental period and became significantly higher than that at the beginning of experiment at day 40 (Fig. 4A–C). At the same time, the average number of CFU‐GEMM colonies during this period was decreased by a factor of ~2.1 (Fig. 4D). In MOG‐treated mice the number of three types of cells was decreased at 9–20 days after injection. Maximal decrease in comparison to the beginning of the experiment (zero time) was: BFU‐E (~1.9‐fold), CFU‐GM (~1.6‐fold) and CFU‐GEMM (~6.5‐fold). Thereafter, cell numbers increased significantly again. Finally at 40 days, the average number of three types of colonies, but not for CFU‐E, was higher in untreated compared to treated mice: BFU‐E (~1.4‐fold), CFU‐GM (~1.2‐fold) and CFU‐GEMM (~1.2‐fold) colonies. In contrast to the early erythroid colonies (BFU‐E), the average content of the late erythroid colonies (CFU‐E) was increased at 9 days after treatment with MOG in comparison to day 0 (6.5‐fold) as well as untreated mice at 9 days (4.0‐fold) and there was no significant change in cell numbers up to 40 days, when their relative amount was 1.8‐fold higher than in untreated control mice. Figure 4E demonstrates the relative content of different types of colonies and a sum of all cell numbers at the beginning (zero time) and at the end (40 days) of the analysis. It is obvious that very different changes in the profiles of differentiation and levels of proliferation of bone marrow progenitors occurred in untreated and treated mice.

### Lymphocyte proliferation in different mouse organs

**Figure 5:**
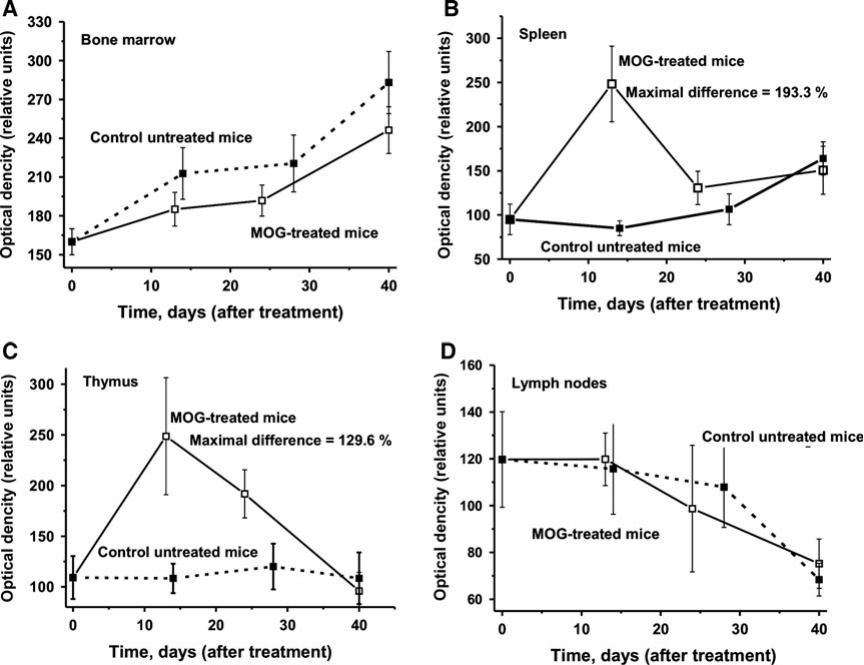
In time changes in lymphocyte proliferation (optical density) in bone marrow (**A**), spleen (**B**), thymus (**C**) and lymph nodes (**D**); in the case of untreated and MOG‐treated mice average values corresponding to seven mice of each group is given. The error in the lymphocyte determination from three independent experiments in the case of every mouse of each group did not exceed 7–10%. For other details, see Materials and methods.

Next we analysed relative levels of lymphocyte proliferation in different organs of mice (Fig. 5). Interestingly, in untreated mice lymphocyte proliferation in bone marrow and spleen slowly increased, and at day 40, it was statistically significantly higher than at day 0 (1.7‐fold for spleen and 1.8‐fold for bone marrow). For thymus, the number of cells was rather constant over time in untreated animals. The increase in lymphocyte proliferation in bone marrow of untreated and treated mice was comparable in the two groups. Following MOG treatment, there was a significant increase in lymphocyte proliferation in the spleen and thymus at 8–20 days after injection (the maximal, statistically significant increase was about 130–193%; Fig. 5B and C). Later, the level of the proliferation in these organs decreased again and was nearly the same for treated and untreated mice at 40 days thereby resembling the numbers at time 0. For lymph nodes, there was a statistically significant 1.8‐fold decrease in lymphocyte proliferation observed in untreated mice at day 40, which was comparable to the decrease found in treated mice.

### Cell apoptosis assay

**Figure 6:**
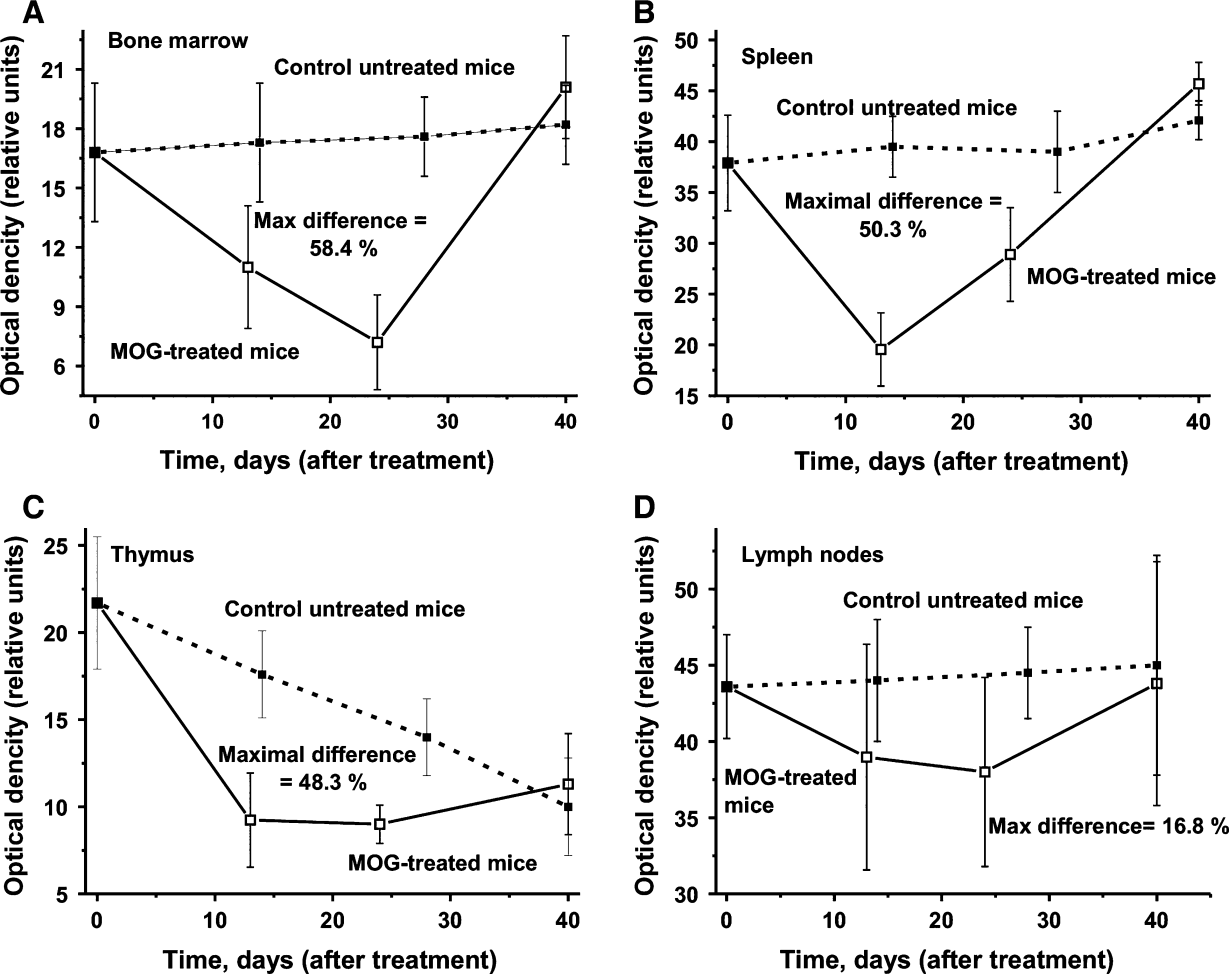
In time changes in cell apoptosis (optical density) in bone marrow (**A**), spleen (**B**), thymus (**C**) and lymph nodes (**D**); in the case of untreated and MOG‐treated mice average values corresponding to seven mice of each group is given. The error in the cell apoptosis determination from two independent experiments in the case of every mouse of each group did not exceed 7–10%. For other details, see Materials and methods.

In the following, we analysed relative levels of cell apoptosis in different organs of mice (Fig. 6). In the case of control untreated mice a tendency of slow increase in cell apoptosis during 40 days was observed in bone marrow, spleen and lymph nodes, while the level of cell apoptosis in thymus was slowly decreased and after 40 days, it was statistically significantly 2.2‐fold lower than at day 0 of the experiment (Fig. 6). Interestingly, maximal decrease in cell apoptosis in all organs of MOG‐treated mice (Fig. 6) occurs at the same time (10–25 days after treatment) as the maximal increase in abzyme activities (Fig. 3) and changes in cell differentiation and proliferation (Figs 4 and 5). In all organs analszed, a statistically significant decrease in average level of cell apoptosis (up to 16.8–58.4%) was observed at 10–25 days after the treatment with MOG (Fig. 6).

## Discussion

Here we analysed, for the first time, changes of a subset of biochemical markers of EAE, namely abzyme RAs, HSC differentiation profile, apoptosis and lymphocyte proliferation in several organs following MOG treatment in autoimmune‐prone C57BL/6 mice. It was notable that untreated C57BL/6 mice revealed a rather high average level of proteinuria (7.2 mg/ml) at 3 months of age, which further increases 1.7‐fold (12 mg/ml) by day 40 (Fig. 1B). Also an increase in MOG‐specific Abs was observed in untreated mice over time. The treatment of mice with MOG promotes this increase in proteinuria approximately twofold (21.8 mg/ml) by day 40 (Fig. 1B). Furthermore, significant increases were observed in the relative content of anti‐DNA and anti‐MOG Abs, activities of abzymes hydrolysing these substrates (Figs 1 and 3), and changes in the relative concentrations of various cells in different mouse organs (Figs 4 and 5) following EAE induction. The spontaneous increase in proteinuria and MOG‐specific Abs in untreated mice suggests that C57BL/6 mice represent a prototypical autoimmune‐prone strain, in which the spontaneous development of autoimmune processes may occur and the state of ‘health’ may be considered provisional. Indeed the mouse SLE pathology is spontaneous, and autoimmune reactions leading to severe pathology develop gradually 34, 35, 36. At 2–3 months of age, MRL‐lpr/lpr mice are usually conditionally healthy; they do not demonstrate proteinuria or other immunological or biochemical indexes of detectable SLE 34, 35, 36. Non‐autoimmune, and even healthy autoimmune MRL‐lpr/lpr mice (aged 2–3 months) are usually characterized by low concentrations of protein in the urine (0.38 mg/ml), and for BALB and CBA mice (0.1–0.12 mg/ml), protein is not increased for a very long time, while proteinuria (≥3 mg/ml) is usually considered to be a good marker of strong autoimmune processes in autoimmune mice (34, 35, 36 and references therein). The first symptoms of spontaneous SLE development, including a 1.5‐ to 2.0‐fold increase in proteinuria, may be revealed in some pre‐diseased MRL‐lpr/lpr mice at 3–5 months of age, and all mice demonstrate all signs of severe SLE at approximately ≥5–7 months of age 34, 35, 36.

It is known that the sera of healthy human beings and mammals contain auto‐Abs to many different antigens, including DNA, and they are highly variable 10, 11, 12, 13, 14, 45. For example, the average concentration of anti‐DNA Abs for non‐autoimmune CBA and BALB mice is 0.03–0.04 A_450_ units, while for healthy MRL‐lpr/lpr mice, this is 0.032 A_450_ units; the latter increases after the spontaneous development of SLE to 0.2 A_450_ units 36. In contrast to MRL‐lpr/lpr, untreated C57BL/6 mice demonstrated an approximately 3.5‐fold higher concentration of anti‐DNA Abs (0.12 ± 0.04 A_450_ units) at 3 months of age (day 0). The change in anti‐DNA Abs for untreated C57BL/6 mice during 0–40 days was statistically insignificant (Fig. 1C). Interestingly, the blood plasma of MS patients contains anti‐DNA Abs at a higher concentration (0.22 A_450_ units) compared to healthy controls and other neurological diseases of non‐autoimmune origin 11, 14. MOG immunization of C57BL/6 mice led to a near‐linear statistically significant twofold increase in anti‐DNA Ab concentration during 30 days (0.24 A_450_ units), and then a further increase to 0.42 A_450_ units, which is higher than that for spontaneous disease (0.2 A_450_ units), but lower than that for MRL‐lpr/lpr mice immunized with DNA (0.6 A_450_ units) 36.

Healthy human donors demonstrate a relative average index of anti‐MBP of 0.08 ± 0.04 A_450_, which is approximately fourfold lower than that for MS patients (0.32 ± 0.08 A_450_ units) 24. Interestingly, the concentration of anti‐MOG Abs in the sera of control (untreated) C57BL/6 mice showed a near‐linear statistically significant ~4.8‐fold increase during 40 days (from 0.023 to 0.11 A_450_ units). After treatment of mice, there was a significant increase in the titre at 10 days (from 0.023 to 0.083 A_450_ units), followed by a plateau phase and a further increase to 0.17 A_450_ units, which was <1.5‐fold higher than for control mice (Fig. 1D). These data indicate that MOG treatment stimulates the formation of Abs to both MOG and DNA in mice. At the same time, the relative titres of Abs to different antigens are not always good indicators of the real development of autoimmune diseases, since the relative titres to autoantigens in healthy human beings and mammals can vary over a very wide range 10, 11, 12, 13, 14, 45.

As we have shown previously, the detection of Abs with DNase activity in human serum may be considered to be a good indicator for the beginning of, or a significant development in, autoimmune reactions associated with several autoimmune pathologies 10, 11, 12, 13, 14. In addition, like in autoimmune patients, only IgG DNase and ATPase activities can be considered statistically significant indicators of pre‐disease conditions of spontaneous SLE in autoimmune prone MRL‐lpr/lpr mice 34, 35, 36. It should be mentioned, that detectable levels of DNase and ATPase activities of MRL‐lpr/lpr mice IgGs can sometimes be revealed 1–2 months earlier than a statistically reliable increase in anti‐DNA Ab concentrations, as well as detection of visual and biochemical signs of mouse SLE 34, 35, 36.

We have measured the relative DNase and protease activity of IgGs from MOG‐treated and untreated mice (Fig. 3). Surprisingly, and in contrast to MRL‐lpr/lpr mice, detectable levels of DNA‐, MOG‐ and MBP‐hydrolysing IgGs in the blood plasma of C57BL/6 mice were found even at the beginning of the experiment (at 3 months of age), and increased near‐linearly; after 40 days, they were statistically significantly higher than at day 0: DNase (6.1‐fold), MOG‐hydrolysing (1.8‐fold), MBP‐hydrolysing (2.8‐fold). Interestingly, the average relative DNase activity during 40 days increased 6.1‐fold (Fig. 3), while there was no statistically significant increase in the titres of anti‐DNA Abs (Fig. 2). This finding is not surprising, since we have previously shown that the relative content of abzymes against different antigens in the total pools of Abs is usually ≤0.01–3% 10, 11, 12, 13, 14. Therefore, the production of a specific small fraction of DNase abzymes may have no detectable influence on the total concentration anti‐DNA Abs. At the same time, the increase in the concentration of total anti‐MOG Abs between day 10 and 28 is faster in untreated compared to treated mice while MOG‐hydrolysing activity is steeply enhanced only in treated animals (Fig. 3E). Therefore, one cannot exclude that the spontaneous production of anti‐MOG Abs without catalytic activity in control mice may be more intensive than MOG‐hydrolysing abzymes.

Furthermore, increased DNA‐, MOG‐ and MBP‐hydrolysing activities of IgGs were detectable at a time corresponding to the beginning and acute phase of EAE (6–8 days), followed by a statistically significant enhancement of their activities at 14 days, and maximal increases at 22 days after MOG treatment (Fig. 3). Later, there was a significant decrease in the activities at the transition from the acute (18–22 days) to the severe chronic phase of EAE (40 days) induced by MOG. Thus, there was a spontaneous and MOG‐induced formation of abzymes hydrolysing DNA, MOG and MBP with high activity in C57BL/6 mice. What may be the reason for this peculiar time course?

It is known that apoptotic cells are the primary source of antigens and immunogens in SLE which triggers the recognition, perception, processing and/or presentation of apoptotic autoantigens by antigen‐presenting cells, and can cause autoimmune processes 31. DNase abzymes from SLE 23 and MS patients 14, and DNA‐hydrolysing Bence‐Jones proteins from multiple myeloma patients 46 are cytotoxic, cause nuclear DNA fragmentation, and induce apoptosis. A significant decrease in apoptosis can be an important factor providing the increased level of specific lymphocytes producing auto‐Abs and abzymes, which are normally eliminated in different organs in mammals.

In Figure 6, it is demonstrated that the level of apoptosis in different organs of untreated mice during spontaneous EAE has a tendency to increase, but varies slightly. However, MOG treatment stimulated a significant decrease in apoptosis in bone marrow, spleen, thymus and lymph nodes; the maximal decrease in apoptosis (16.8–58.4%) corresponds to the beginning and acute phase of EAE (8–22 days; Fig. 6) induced by MOG. Later, the level of apoptosis in treated mice increased and became comparable with that of untreated mice (Fig. 6). In addition, the time of maximal increase in lymphocyte proliferation in thymus and spleen (Fig. 5) correlated well with the maximal decrease in apoptosis (Fig. 6). A specific situation was observed for lymph nodes, which revealed the lowest level of decrease in apoptosis as well as a decrease in lymphocyte proliferation in treated and untreated mice (Figs 5 and 6). The decrease in apoptosis in different mouse organs can lead to an increase in proliferation of not only lymphocytes typical for healthy mammals (Fig. 5), but also specific lymphocytes that are harmful and usually eliminated by apoptosis in different organs of mammals. DNA‐hydrolysing Abs are harmful to humans and mammals; they stimulate apoptosis, which leads to an increase in the concentration of cell components, including DNA, in the blood and to a strengthening of the autoimmune processes 9, 10, 11, 12, 13, 14. Similar to anti‐MBP abzymes of MS and SLE patients 14, abzymes of mice hydrolysing MOG and MBP can destroy the myelin‐proteolipid sheath of axons.

The situation in the case of bone marrow was a specific one. While the decrease in apoptosis in bone marrow was maximal at 24 days after MOG treatment (Fig. 6A), the level of lymphocyte proliferation constantly increased up to day 40 of the analysis (Fig. 5A). These findings indicate that very specific immune processes may be occurring in the bone marrow and other organs of autoimmune‐prone mice. During the spontaneous development of a severe SLE‐like pathology, a specific reorganization of the immune system of MRL‐lpr/lpr mice first leads to conditions associated with a production of Abs hydrolysing DNA, ATP and polysaccharides, with low catalytic activities (conditionally pre‐diseased mice) 36. A significant increase in DNase, ATPase and amylase IgG RAs associated with a transition from pre‐diseased to severely diseased states is correlated with additional changes in the differentiation of mice bone marrow HSCs and lymphocyte proliferation in different organs. After immunization of healthy control mice with DNA, the production of abzymes was also observed, but compared with spontaneously pre‐diseased and diseased mice, there was no significant change in the HSC differentiation profile compared with untreated mice 36. However, an increased level of lymphocyte proliferation in thymus, spleen and lymph nodes, and a significant suppression of apoptosis in these organs were observed 36.

Since, similar to MRL‐lpr/lpr, C57BL/6 mice also reveal features of autoimmune‐prone mice, it was reasonable to expect similar changes in the HSCs of these mice before and after MOG immunization. Figure 4 revealed that following a slight initial drop, there was a constant increase in the relative amount of BFU‐E (Fig. 4A), CFU‐GM (Fig. 4B) and CFU‐E (Fig. 4C) in untreated mice over time. Only the CFU‐GEMM colony had a constant decrease (Fig. 4D). Following treatment of mice with MOG, there was a significant decrease in BFU‐E and CFU‐GM while the number of CFE‐E colonies was increased (Fig. 4). Interestingly, a significant decrease in the CFU‐GEMM units was observed at 20 days in comparison with untreated mice (Fig. 4D). All observed changes in the number of these colonies (Fig. 4) occurred at the same time and in parallel with the decrease in apoptosis in bone marrow (Fig. 6A). Finally, at day 40 after immunization, remarkable changes in the relative number of cells in the bone marrow cell differentiation profile (relative amount of cells of different types) were observed for treated and untreated mice compared with control mice at day 0.

An ever‐increasing number of observations suggests that autoimmune diseases originate from defects in the HSCs 36, 37. Non‐autoimmune‐prone CBA mice did not demonstrate any remarkable changes in bone marrow cell differentiation profile during at least 7 months of age 36. The relative content of total erythroid cells [BFU‐E+ CFU‐E (28.4%), CFU‐GM (69.2%) and CFU‐GEMM (2.4%)] in the case of CBA mice may be considered as an example of a typical ratio of different HSCs in healthy mice. First, we tried to compare the relative content of total erythroid cells, CFU‐GM and CFU‐GEMM at 3 months of age in healthy, non‐autoimmune CBA and conditionally healthy, autoimmune‐prone MRL‐lpr/lpr, with C57BL/6 mice (Fig. 4F). The relative content of total erythroid cells in the case of conditionally healthy MRL‐lpr/lpr (46.4%) and C57BL/6 (60.5%) was statistically significantly higher (1.6‐fold and 2.1‐fold, respectively) than that for CBA mice (28.4%) (Fig. 4F). At the same time, the relative content of CFU‐GM cells at 3 months of age in MRL‐lpr/lpr (50.0%) and C57BL/6 (34.2%) mice was significantly lower (1.4‐fold and 2.0‐fold, respectively) than that for CBA mice (69.2%). Diseased MRL‐lpr/lpr and C57BL/6 mice, after the induction of EAE, demonstrated a significant additional increase in the relative content of total erythroid cells (69–71%), while the content of CFU‐GM cells (20.2–27.4%) was additionally decreased (Fig. 4F). Interestingly, the relative content of total erythroid (70.9% and 71.1%) and CFU‐GM cells (27.4% and 27.5%) before and after MOG treatment in C57BL/6 mice was the same (Fig. 4F). Conditionally healthy MRL‐lpr/lpr (3.6%) and C57BL/6 (5.6%) at 3 months of age were characterized by a 1.5‐ and 2.3‐fold, respectively, higher content of CFU‐GEMM cells compared with CBA mice (2.4%). Transition from conditionally healthy (3.6%) to spontaneous SLE (10.7%) of MRL‐lpr/lpr leads to an increase in the content of CFU‐GEMM cells by a factor of ~3.0. At the same time, transition from conditionally healthy (5.6%) either to spontaneously diseased or MOG‐treated C57BL/6 mice (1.5%) leads to a 3.7‐fold decrease in CFU‐GEMM cells (Fig. 4F). Thus, the spontaneous development of SLE by MRL‐lpr/lpr and induction of EAE in C57BL/6 is characterized by very similar changes in the relative content of the total erythroid and CFU‐GM cells, while the relative content of CFU‐GEMM cells is changed in different directions. Taken together, autoimmune‐prone MRL‐lpr/lpr and C57BL/6 are characterized by the spontaneous development of a condition, which is susceptible for autoimmune attacks and associated with significant, and in many respects similar changes in the differentiation of bone marrow HSCs, lymphocyte proliferation in different organs and production of abzymes hydrolysing DNA. In contrast to MRL‐lpr/lpr mice, the induction of EAE in C57BL/6 mice correlates with production of abzymes hydrolysing MOG and MBP. Most probably, the development of other different autoimmune diseases is also associated with significant changes in the HSC differentiation profile and lymphocyte proliferation, which may be an important prime cause for the initial stage of these autoimmune processes.

## Conflicts of interest

The authors declare no conflicts of interest.

## Author contribution

The author(s) have made the following declarations about their contributions: conceived and designed the experiments: IAO, NAP, VNB and GAN; performed the experiments: VBD, TAP, AK, LBT, JAL, AAA and SVS; analysed the data: GAN, TB, SGM, IAO and NAP; contributed reagents/materials/analysis tools: IAO, NAP, VNB, TB and SGM; wrote the paper: GAN, TB, SGM and IAO; developed the theoretical description: GAN, TB and SGM.
